# The Critical Role of Nurr1 as a Mediator and Therapeutic Target in Alzheimer’s Disease-related Pathogenesis

**DOI:** 10.14336/AD.2019.0718

**Published:** 2019-07-18

**Authors:** Seong Gak Jeon, Anji Yoo, Dong Wook Chun, Sang Bum Hong, Hyunju Chung, Jin-il Kim, Minho Moon

**Affiliations:** ^1^Department of Biochemistry, College of Medicine, Konyang University, Daejeon, 35365, Republic of Korea; ^2^Department of Core Research Laboratory, Clinical Research Institute, Kyung Hee University Hospital at Gangdong, Seoul 05278, Republic of Korea; ^3^Department of Nursing, College of Nursing, Jeju National University, Jeju-si 63243, Republic of Korea

**Keywords:** Alzheimer’s disease, Nurr1, NR4A2, memory, neuroprotection, neuroinflammation

## Abstract

Several studies have revealed that the transcription factor nuclear receptor related 1 (Nurr1) plays several roles not only in the regulation of gene expression related to dopamine synthesis, but also in alternative splicing, and miRNA targeting. Moreover, it regulates cognitive functions and protects against inflammation-induced neuronal death. In particular, the role of Nurr1 in the pathogenesis of Parkinson’s disease (PD) has been well investigated; for example, it has been shown that it restores behavioral and histological impairments in PD models. Although many studies have evaluated the connection between Nurr1 and PD pathogenesis, the role of Nurr1 in Alzheimer’s disease (AD) remain to be studied. There have been several studies describing Nurr1 protein expression in the AD brain. However, only a few studies have examined the role of Nurr1 in the context of AD. Therefore, in this review, we highlight the overall effects of Nurr1 under the neuropathologic conditions related to AD. Furthermore, we suggest the possibility of using Nurr1 as a therapeutic target for AD or other neurodegenerative disorders.

## 1. The nuclear receptor related-1 protein, Nurr1

The nuclear receptor related 1 (Nurr1) protein, also known as nuclear receptor subfamily 4, group A, member 2 (*NR4A2*). Nurr1 belongs to the nuclear receptor subfamily 4A (NR4A), which consists of *NR4A1*, *NR4A2*, and *NR4A3*, also known as Nur77, Nurr1, and Nor1, respectively [1, 2]. Nurr1 is robustly expressed in the central nervous system (CNS) [3, 4]. Similar to other members of the NR4A, Nurr1 has been considered as an orphan nuclear receptor, whose endogenous ligand has not been identified [1]. Nurr1 is well known to play an essential role in the development, function, and maintenance of midbrain dopaminergic neurons [5-7]. In particular, Nurr1 is known to play an integral role in multiple signaling pathways involved in the differentiation and phenotype of dopaminergic neurons [8]. It is also targeted by miRNAs in dopaminergic neurons, and is alternatively spliced by cyclic adenosine monophosphate (cAMP)-responsive element-binding protein (CREB)-regulated transcription co-activators [9, 10]. Notably, a recent report suggesting that CREB-regulated transcription coactivator-1 (CRTC1) mediates expression of the Nurr1 gene provided evidence for specific molecular mechanisms for the regulation of Nurr1 expression in primary cortical neurons [11]. Nurr1 expression is also found to be directly induced by various stimuli such as inflammatory signals. Once Nurr1 is activated by various factors, it binds to specific DNA sequences in the promoter region of the target genes to positively regulate their expression [12].

In addition to the role of Nurr1 in the pathogenesis of dopamine-related neurological disorders [6, 13], several studies have revealed the involvement of Nurr1 in reward-seeking behavior [14], symptoms of schizophrenia [15], and pathogenesis of Alzheimer’s disease (AD) [16]. Although the endogenous ligand of Nurr1 has not yet been identified, the cognition-enhancing effects of Nurr1 agonists, which have been demonstrated in wild-type (WT) and PD mice, support the potential of Nurr1 as a therapeutic target for neurodegenerative disease [17-20].

**Table 1:** Overview of the possible roles of Nurr1 in AD.

	References
Nurr1 expression in AD	• Nurr1 immunofluorescence intensity is reduced in the substantia nigra of AD patients	[13]
	• Nurr1 mRNA levels are reduced in APP_swe, lnd_ mutant mice	[62, 63]
	• The number of Nurr1(+) cells is age-dependently reduced in the subiculum of 5XFAD mice	[90]
	• Nurr1 protein is co-localization with Aβ at the early stage in 5XFAD mice	[90]
	• Nurr1 protein and mRNA are downregulated in Aβ_1-42_ fibril-treated CGNs and the hMSC cell line	[65]
Neuroprotective effects	• MPTP-induced neurotoxic vulnerability of dopaminergic neurons is increased in Nurr1^(+/-)^ mice	[59]
	• Nurr1 in microglia and astrocytes protects neurons by regulating the production of toxic mediators	[79]
	• Ligand and agonist of Nurr1 shows neuroprotective effect against oxidative insult such as MPTP and 6-OHAD	[17, 19, 20]
	• Increased expression of Nurr1 upregulates genes involved in ROS detoxification such as *Sesn3*, *Alb2*, and *Sod1*	[81]
	• In NSCs, the overexpression of Nurr1 protects against oxidative stress by downregulating cell death-related proteins such as caspase-3 and caspase-11	[60]
	• Exogenous Nurr1 induces the differentiation of dopaminergic neurons, and sustained Nurr1 expression improves survival of dopaminergic neurons	[83, 149]
Anti-inflammatory effects	• Nurr1 phosphorylation promotes binding to p65 and recruits the CoREST complex to promoters of inflammatory genes, resulting in inhibition of neuroinflammation	[79]
	• Overexpression of Nurr1 suppresses inflammation, whereas knockdown of Nurr1 enhances inflammation	[16]
	• NR4A receptors are involved in a negative feedback loop as modulators of the inflammation mechanism	[93]
	• Inflammatory stimulus (e.g., LPS) up-regulates Nurr1 mRNA expression in microglia	[96]
Peripheral immune regulation	• Nr4a-TKO mice cannot produce T_reg_ cells and die early due to systemic autoimmunity	[118]
	• Nurr1 induces Foxp3 in CD4+ T cells via modulating histone modifications	[94]
	• Nurr1 can regulate Th17 cell-mediated autoimmune inflammation	[112]
Cell-cycle regulation	• Nurr1 promotes cell-cycle arrest in the G1 phase as well as differentiation of MN9D cells	[134]
	• Overexpression of Nurr1 inhibits proliferation via increased expression of p27Kip1 in VSM cells	[135]
	• Nurr1 overexpression restricts proliferation via upregulated expression of p18 in HS cells	[136]
	• Nurr1 induced after ischemic injury promotes IE cell proliferation via inhibition of p21	[139]
	• Treatment with the Nurr1 agonist increases proliferation via phosphorylation of Akt and Erk1/2 in AHP cells	[18]
Neurogenic effects	• Nurr1 induces neural differentiation of ECP cells through an extrinsic paracrine mechanism	[152]
	• The ventral midbrain in Nurr1 knockout mice shows reduction of NPC differentiation	[150]
	• Nurr1 promotes dopaminergic neuron production and suppresses inflammatory factors	[155]
	• Overexpression of Nurr1 in NPCs obtained from the SVZ of rats induces dopaminergic neurons	[149]
	• The Nurr1 agonist amodiaquine causes a significant increase in adult hippocampal neurogenesis	[18]
Memory-enhancing effects	• Formation of long-term memory in the hippocampus depends on the cAMP/PKA/CREB signaling pathway, which also controls transcription of Nurr1	[48, 168]
	• Inhibition of HDAC increases Nurr1 expression, and enhances memory, which is attenuated by protein suppression, siRNA knockdown, and Nurr1 knockout	[15, 53, 54, 171]
	• Dominant negative Nurr1 mice inhibition of Nurr1 function impairs hippocampal long-term potentiation	[55]
Vascular pathology mitigation	• Overexpression of Nurr1 inhibits vascular lesion via reducing SMCs proliferation and inflammation	[135]
	• Overexpression of Nurr1 reduces oxidized-low-density lipoprotein uptake and inflammatory responses in macrophages	[178]
Role in metabolism	• Abnormal expression of Nurr1 is associated with glucose metabolism and metabolic syndrome	[183, 184]
	• NR4A receptors are induced by metabolic-related stimuli such as fatty acids, glucose and insulin	[185]
	• NR4A receptors including Nurr1 are involved in increased glucose uptake in the skeletal muscle	[186]
Therapeutic potential of Nurr1 activation	• Nuclear receptors serve as a critical mediator of Aβ homeostasis	[203-205]
	• Nurr1 expression can suppress NF-κB signaling pathway	[79]
	• Nurr1 regulates AD-related pathogenesis and cognitive function in 5XFAD mice	[16]

AD: Alzheimer’s disease, Aβ: amyloid beta, APP: amyloid precursor protein, CGNs: cerebellar granule neurons, hMSC: human mesenchymal, NSCs: neuronal stem cells, MN9D cells: dopamine-synthesizing cell line, VSM cells: vascular smooth muscle cells, HS cells: hematopoietic stem cells, IE cells: intestinal epithelial cells, AHP cells: adult hippocampal neural precursor cells, CoREST: co-repressor for RE1 silencing transcription factor, NPCs: neural precursor cells, SVZ: subventricular zone, SMCs: Smooth muscle cells, ECP cells: embryonic cortical precursor cells, HDAC: histone deacetylase, BACE1: beta-secretase 1

## 2. The roles of Nurr1 in AD-related pathology

AD is known to be the most common cause of dementia and is responsible for 60%-70% of the cases of dementia [21, 22]. AD patients exhibit impairment of cognitive functions, which is mediated by abnormal accumulation of amyloid plaques containing amyloid beta (Aβ) and neurofibrillary tangles (NFT) in the brain [23-25]. With the increasing focus on AD over the past century because of the gradual aging of the global population, the pathophysiologies of AD [26-29], as well as its clinical manifestations [30, 31], diagnosis [32, 33], and genetic characteristics [34, 35] are now relatively well understood. Several studies on therapeutic approaches for AD have been performed, including those involving cholinesterase inhibitors, *N-methyl-d-aspartate (NMDA) receptor antagonists* [36-39], and anti-Aβ therapy [40-43]. Nevertheless, there is no disease-modifying therapy yet [44]. Although the exact mechanisms of AD pathogenesis are unclear, intracellular and extracellular Aβ are thought to be major causative factors associated with AD-related pathologies, such as neurodegeneration and cognitive dysfunction [45-47]. Interestingly, Nurr1 is known to act as a critical regulator of hippocampal function, hippocampal synaptic plasticity, and cognitive functions [15, 48-55], and is an essential mediator of neuroprotection or anti-inflammation after exposure to neuropathological stress [19, 56-61]. In addition, a number of studies have indicated altered levels of Nurr1 in Aβ-treated neuronal cells, animal models of AD, and the brains of patients with AD [13, 62-65], implying that Nurr1 may play a role in the pathogenesis of AD. Recent studies have shown that Aβ_1-42_ fibrils not only lead to upregulation of tau hyperphosphorylation and presenilin 1 mRNA, which are hallmarks of AD pathology, but also significantly reduce Nurr1 mRNA levels in an *in vitro* model of AD [65]. Immunofluorescence staining with Nurr1-specific antibody in 5XFAD mice, an animal model of AD, showed that the Nurr1 protein is markedly expressed in the brain areas with Aβ accumulation. Moreover, the number of Nurr1-expressing cells is decreased in 5XFAD mice with AD progression, compared with WT mice [64]. In contrast, the levels of miR-184, which directly targets the 3′ UTR of the *NR4A2* transcript, are reduced in the hippocampus of late-onset AD patients. In addition, expression of *NR4A2* and miR-184 is inversely correlated [66]. These findings suggest that Nurr1 is not only highly implicated in cases of AD, but also can modulate AD pathogenesis. The following sections will discuss the critical roles and effects of Nurr1 in neurodegenerative diseases (Table 1).

### 2.1. Neuroprotective effects of Nurr1

Neuronal death is the main pathogenic factor underlying neurodegenerative diseases such as AD and PD [67, 68]. Under neuropathological conditions, including neuroinflammation, excitotoxicity, and oxidative stress, neurons can be rescued by upregulation or activation of neuroprotective factors such as reactive oxygen species (ROS) scavengers or anti-apoptotic molecules [69-71]. Therefore, induction of neuroprotective factors may be a therapeutic strategy for the treatment of neuro-degeneration-related diseases.

Several studies have shown that Nurr1 has anti-apoptotic or neuroprotective roles against neuropathological stress or insults [56-60, 72-74]. Nurr1 expression is known to be regulated by various stimuli such as inflammatory cytokines, cAMP, and growth factors [75-77]. In comparison with WT mice, neurons from Nurr1 heterozygous mice exhibit greater vulnerability to neurotoxic challenges [59]. In addition, survival of dopaminergic neurons in the midbrain of mice was inhibited by genetic deletion of Nurr1 during development [78]. Moreover, it has been demonstrated that Nurr1 inhibits dopaminergic neuronal loss by suppressing inflammatory stimuli in the microglia and astrocytes [79]. Nurr1 changes its subcellular distribution in response to oxidative stress [80]. In addition, the ligand and agonist of Nurr1 showed neuroprotective effects on subsequent oxidative insult such as MPTP and 6-OHAD [17, 19, 20]. Especially, increased expression of Nurr1 by lentiviruses upregulated genes involved in ROS removal, such as *Sesn3*, *Abl2*, and *Sod1*, and demonstrated that Nurr1 is an essential mediator of CREB-dependent neuroprotection in oxidative stress [81]. Furthermore, Nurr1 overexpression protected neuronal stem cells against oxidative stress through downregulating cell death related protein such as caspase-3 and caspase-11 [60]. A number of studies have demonstrated the protective role and correlation of Nurr1 in cell death by oxidative stress, but the detailed molecular mechanism of Nurr1 against oxidative stress remains unclear [17, 19, 20, 60, 81-83]. Interestingly, a contemporary study reporting the direct binding of dopamine metabolite on Nurr1 and its stimulation of Nurr1 activity may provide an evidence for mechanism underlying the role of Nurr1 in sensing and responding the oxidative stress [84]. Moreover, Jo *et al*. reported that exogenous Nurr1 expression in neural precursor cells (NPCs) induced differentiation of dopaminergic neurons, higher resistance to toxic stimuli, and enhanced survival [83]. Although there has been only a few reports regarding the direct roles of Nurr1 in neuronal death in AD, interestingly, it has been demonstrated that Nurr1 is not only involved in the protection of dopaminergic neurons but also of GABA-positive neurons *in vitro* [79]. Since the levels of GABAergic neurotransmission as well as GABAergic signaling are significantly altered in AD [85, 86], it can be speculated that strategies for preserving GABAergic neurons by maintaining Nurr1 expression. All these reports suggest that Nurr1 may have neuroprotective effects against the pathogenesis of neurodegenerative diseases. A recent study has reported the protective role of Nurr1 in neuronal death in AD [16].

### 2.2. Anti-inflammatory effects of Nurr1

Neuroinflammation is one of the most important aspects of AD pathogenesis. Although resting glial cells maintain the microenvironment in the brain, activated glia contributes to neuronal damage by releasing neurotoxic molecules [87, 88]. Over-activated microglia and astrocytes release several detrimental compounds such as ROS, superoxide (O_2_^•-^), nitric oxide (•NO), and cytokines, which cause neuronal damage. The Aβ peptide is known to directly activate microglial cells, and activated cells are recruited around Aβ plaques before symptom development [89]. In addition, our previous studies revealed that Aβ oligomers (AβO) may induce both gliosis and neurodegeneration in the animal brain [90-92]. A significant increase in the number of microglial cells and decrease in the number of neurons were simultaneously observed in the brain of AβO-injected mice [90-92]. NR4A receptors are promptly activated by inflammatory stimuli, thus regulating not only initiation of inflammatory responses but also in the late stages of inflammation. NR4A receptors are involved in a negative feedback loop as modulators of inflammation [93]. Nurr1 can mediate inflammatory responses and regulate the function of immune cells [94, 95]. In microglia, inflammatory stimuli such as lipopolysaccharides (LPS) up-regulate Nurr1 mRNA expression [96]. Notably, Nurr1 shows potent anti-inflammatory activity in the CNS. In microglia and astrocytes, Nurr1 receptors inhibit the expression of pro-inflammatory cytokines, which are neurotoxic and eventually induce neuronal death, whereas reduction of Nurr1 enhances the inflammatory responses [79]. Nurr1 mediates the GSK3β-dependent repression of nuclear factor kappa-light-chain-enhancer of activated B cells (NF-κB). Mechanistically, inflammatory signals induce Nurr1 phosphorylation and sumoylation, thus promoting Nurr1 binding to p65 and recruitment of the co-repressor for the RE1 silencing transcription factor (CoREST) complex to promoters of inflammatory genes, resulting in modulation of neuroinflammation [79]. Interestingly, a previous study revealed that deletion of amino acids 1-31 from the N-terminal region of Nurr1 yields better performance in transcription compared to full-length Nurr1 [97]. In addition, a recent study has provided direct evidence that modulation of Nurr1 can be involved in Aβ-mediated neuroinflammation [16]. In conclusion, Nurr1 can serve as a possible therapeutic target for treatment of AD by inhibiting the transcription of inflammatory genes and modulating the function of immune cells.

### 2.3. Peripheral immune cell modulation of Nurr1

The early stage of AD involves both the activation of microglia and astrocytes overexpressing cytokines around the Aβ plaques and an increase in the levels of pro-inflammatory cytokines in the peripheral blood [98]. In addition, the cross-talk between the peripheral blood and the brain via a damaged blood-brain barrier (BBB) may be enhanced in AD patients, thereby contributing to neuroinflammation in AD [99]. As evidenced, an increase in the number of T cells was observed in the brain parenchyma of AD patients [100]. Several lines of evidence have indicated that modulation of T helper (Th) cells may be involved in AD pathologies [101-104]. In particular, CD4 and CD8 T cells specifically migrated to the Aβ plaques, thus enhancing elimination of Aβ plaques [105]. Moreover, immunization with the A*β*_42_ DNA trimmer was shown to suppress antigen-specific Th17 and Th1 cell proliferation [106].

Peripheral blood mononuclear cells, CD4+ T cells and monocytes obtained from patients with multiple sclerosis (MS) have been reported to have decreased Nurr1 gene expression [107, 108]. In addition, microarray analysis showed that key nuclear receptor family genes such as *NR4A1 *(Nur77) and *NR4A2 *(Nurr1), which are important for the nuclear receptor-dependent apoptosis in the peripheral blood of the pre-disease state in MS patients, were suppressed [109]. In contrast, over-expression of Nurr1 was observed in peripheral blood T cells derived from relapsing-remitting MS patients [110]. In an experimental autoimmune encephalomyelitis (EAE) model that serves as an animal model of MS, effector T cells infiltrated the parenchyma of the CNS [111]. Nurr1 is selectively over-expressed in T cells in the peripheral blood whereas expression of Nurr1 in the T cells in lymphoid organs did change during the induction of EAE. In addition, interleukin (IL)-17-producing tyrosine hydroxylase (TH)-positive cells express Nurr1 regardless of interferon (IFN)-γ secretion [112]. Hence, since Nurr1 could be a useful biomarker for determining the status of T cells in MS [113], assessment of Nurr1 expression in T cells in AD could also be useful to identify changes in T cell activation status. In addition, heterozygous Nurr1 mice promoted early onset of EAE and increased the infiltration of inflammatory cells into the spinal cord [114], indicating that Nurr1 is involved in the pathophysiology of autoimmune diseases such MS. Consequently, Nurr1 could be an innovative therapeutic target for various autoimmune diseases. In human inflammatory joint disease, Nurr1 has been identified as a molecular target of methotrexate (MTX)-related reactions. MTX considerably subdues Nurr1 expression in patients with active psoriatic arthritis. In the synovial tissue, MTX selectively regulates Nurr1 induced by inflammatory stimulation and also modulates expression of growth factors in resident cells. Moreover, suppressive effect of MTX on Nurr1 expression is mediated by adenosine release [115].

Because Nr4a receptors play an important role in initiating regulatory T (T_reg_) cell development in the thymus [94, 116, 117], the role of Nr4a receptors in peripheral immune regulation has been examined in Nr4a-triple-knockout (Nr4a-TKO) mice, Nur77 (*Nr4a1*)^-/-^, Nurr1 (*Nr4a2*)^-/-^, and Nor-1 (*Nr4a3*)^-/-^. The Nr4a-TKO mice could not produce T_reg_ cells and died early due to systemic autoimmunity [118]. Specifically, Nurr1 binds directly to the Foxp3 promoter, leading to activation of transcription and the development of T_reg_ cells. In addition, Nurr1 has been reported to bind directly to the regulatory regions of Foxp3, at which Nurr1 intervenes via histone modifications. Furthermore, in Nurr1-deficient T cells, aberrant Th1 induction is increased but T_reg_ cell induction is rather decreased [94]. In conclusion, Nurr1 plays central roles not only in regulating the induction and suppressive functions of T_reg_ cells but also in inhibiting aberrant Th1 induction. Moreover, Nurr1 can regulate the Th17 cell-mediated autoimmune inflammation, contributing to the pathogenesis of MS, an immune disease of the nervous system [112]. Since peripheral immune functions are involved in the pathogenesis of AD [119, 120], modulation of peripheral immune responses though Nurr1 may be a potential therapeutic strategy against AD.

### 2.4. Cell-cycle regulation of Nurr1 and AD

Neurons are generally considered as postmitotic cells, and can cell undergo cell cycle re-entry in neurodegenerative conditions [121, 122]. Basal forebrain and hippocampal pyramid neurons in the brain with AD have been reported to progress from the G1-phase to the S-phase [123]. In brains with AD and mild cognitive impairment, the expression levels of markers associated with cell cycle and proliferation, such as the proliferating cell nuclear antigen (PCNA), cyclin D, and B1, are increased in various regions including the entorhinal cortex, hippocampus, and nucleus basalis of Meynert [124]. Moreover, the presence of active cdc2 and cyclin B_1_ complex was observed in the AD brain tissue [121]. In addition, senescence-accelerated mice-prone 8, which show the major pathologic features of AD such as Aβ accumulation and tau phosphorylation, not only show enhanced CDK5 and GSK3β expression, but also show increased expression of various cell-cycle re-entry markers such as CDK2, cyclins A, D1, E, and B [125]. There are several reports on the correlation between cell-cycle-related kinases and histological hallmarks of AD. p25, a truncated form of the subunit p35 that activates CDK5, is not readily degraded and is found to accumulate in the brain of patients with AD. Subsequently, the formation of the p25/CDK5 complex induced tau hyperphosphorylation and apoptosis [126]. In addition, soluble Aβ oligomers also promoted neuronal cell-cycle re-entry via phosphorylation of tau [127]; cell-cycle progression through CDK5 and CDC2 kinases induced phosphorylation of the amyloid precursor protein (APP) [128-130], and phosphorylation of APP facilitated Aβ generation [131]. Moreover, a broad promotion of the cell cycle in the AD brain leads to a mitotic catastrophe, which is the result of dysregulated or failed mitosis, suggesting that this may be one of the mechanisms of neuronal death in AD [132, 133].

The role of Nurr1 in the cell cycle has been suggested after assessment of cell-cycle-related molecules in various cells. In the dopamine-synthesizing cell line (MN9D cells), Nurr1 promoted cell-cycle arrest in the G1 phase as well as morphological differentiation, and these effects did not require the formation of heterodimers with retinoid X receptors (RXR) [134]. In vascular smooth muscle cells, lentivirus-mediated Nurr1 overexpression inhibited proliferation with increased expression of the crucial cell-cycle inhibitor p27^Kip1^ which induces G1 cell-cycle arrest [135]. Similarly, in hematopoietic stem cells, Nurr1 overexpression restricted cell proliferation by upregulating the expression of p18, which inhibits the cyclin D/CDK4/6 complexes required for cell-cycle progression in G1-phase [136]. In addition, the mechanism by which Nurr1 promotes migration and inhibits proliferation in mesenchymal stem cells (MSCs) may involve the ability of Nurr1 to reduce the percentage of cells in the S-phase [137]. Furthermore, overexpression of Nurr1 in olfactory bulb stem cells induces cell-cycle exit, inhibits proliferation, and induces a TH neuronal fate mediated by Fgfr2 expression [138]. In contrast, Nurr1 induced after intestinal ischemia/reperfusion injury promoted proliferation of intestinal epithelial cells via inhibition of p21^Waf1/cIP1^ gene transcription [139]. Moreover, in the mouse hippocampus and adult hippocampal neural precursor cells, pharmacological stimulation of Nurr1 with a Nurr1 agonist resulted in increased proliferation as well as phosphorylation of Akt and Erk1/2 [18].

These results suggest that Nurr1 may interfere negatively or positively with the cell cycle depending on the cell type and its environment. They also suggest that Nurr1 may have a positive effect on AD by promoting proliferation of neural stem cells or by suppressing the abnormally promoted cell cycle in the AD brain. However, the correlation of Nurr1 with the cell cycle in AD has not yet been directly reported.

### 2.5. Neurogenic effects of Nurr1 in the adult brain

Neurons are generated and differentiated from neural stem cells in the adult brain. This process is called adult neurogenesis, and takes place mainly in two brain regions, the subgranular zone of the hippocampal dentate gyrus (SGZ) and the subventricular zone of the lateral ventricle (SVZ) [140, 141]. In particular, adult hippocampal neurogenesis at SGZ regulates learning and memory functions by generating newborn neurons derived from neural stem cells [140, 142, 143]. Studies have shown that altered hippocampal neurogenesis occurs in the early stage of AD even prior to pathologic changes [144]. Several key molecular players involved in AD pathogenesis have been found to regulate hippocampal neurogenesis [144-148].

A recent study demonstrated that treatment with the Nurr1 agonist amodiaquine (AQ) in mice significantly contributed to enhanced adult hippocampal neurogenesis, resulting in enhancement of cognitive function. Moreover, knockdown of Nurr1 inhibited proliferation of adult hippocampal neural stem cells [18]. In addition, overexpression of Nurr1 in NPCs isolated from the SVZ of adult rats resulted in functional dopaminergic neurons. Transplantation of Nurr1-induced dopaminergic neurons lead to differentiation and integration *in vivo*, and improved the behavioral disorders of parkinsonian rats [149]. Furthermore, *in vivo* studies showed that Nurr1-deficient mice exhibited deficits in the differentiation of dopaminergic neurons in the ventral midbrain [78, 150]. In support of this notion, Nurr1 is not only well known to play a key role in the differentiation and maturation of dopaminergic neurons [151-155] but may also exert an important role in neurogenesis [156]. These studies suggest that Nurr1 may contribute to the rescue of impaired adult neurogenesis in AD. Indeed, a recent study has shown that administration of the Nurr1 agonist AQ can reverse impaired neuronal fate specification of hippocampal neural stem cells in Aβ-overexpressing mice [16].

### 2.6. Memory-enhancing effects of Nurr1

Aβ is known to be a major contributor of memory impairment in AD, and results in cognitive deficits by inducing neuroinflammation, neuronal death, inhibition of synaptic transmission, synaptic loss, and impairment of adult neurogenesis [88, 157-159]. Furthermore, under AD conditions, there are other pathways that cause cognitive dysfunction and memory decline, such as abnormal activity of the NMDA receptor [160, 161]. Recent studies have suggested that Nurr1 may play a role in the regulation of cognitive function, since hippocampus-dependent memories were impaired in Nurr1 knockdown or NR4A dominant-negative transgenic mice [48, 49, 54] and an increase in Nurr1 mRNA expression was observed when mice were submitted to spatial learning tasks [50]. Thus, it is important to investigate whether Nurr1 has a direct influence on AD-related cognitive functions.

Several studies have discovered that Nurr1 regulates learning and memory functions [15, 49-55]. In many systems, the CREB signaling pathway is important for transcription of memory-related genes [162], and this pathway controls transcription of Nurr1 [163]. Additionally, long-term memory in the hippocampus depends on CREB-related pathways that can regulate Nurr1 expression [48, 164-168]. Several studies demonstrated that behavioral task training increases Nurr1 gene expression in subregions of the hippocampus [50, 53, 54]. Moreover, a network analysis of genes in the dentate gyri of long-term potentiation-induced rats revealed that expression of NR4A nuclear receptors, including Nurr1, was upregulated [169]. Expression of Nurr1 in cultured hippocampal neurons is revealed to be increased after treatment with GABA antagonists [170].

Inhibition of histone deacetylase (HDAC) by trichostatin A increases Nurr1 expression, and enhances memory [171]. Similarly, enhancement of memory by HDAC inhibition is attenuated and memory enhancement is impaired by negative protein expression of Nr4a family receptors [54]. Moreover, memory functions are impaired by siRNA knockdown of Nr4a2 [53] and generation of heterozygosity for the Nurr1 gene [15]. A recent study also revealed the importance of the NR4A family, including Nurr1, showing that hippocampal long-term potentiation was impaired in dominant-negative Nr4a transgenic mice [55]. Interestingly, although the precise mechanism for cognitive enhancement is unclear, one placebo-controlled study showed that the intermittent preventive administration of AQ, an antimalarial agent and Nurr1 agonist, enhanced cognitive performance in semi-immune schoolchildren [172]. Furthermore, a recent study has demonstrated that administration of the Nurr1 agonist AQ restored damaged spatial working memory in Aβ-overexpressing mice [16]. However, how Nurr1 affects AD patients with memory failure has not been examined, and we anticipate that further studies on Nurr1 will uncover its importance on cognitive functions in brains with AD.

### 2.7. The role of Nurr1 in vascular pathologies

Cerebral amyloid angiopathy (CAA) is an AD-related histopathology showing pathological changes such as deposits of Aβ in the blood vessels of the CNS and the walls of leptomeningeal arteries [173]. CAA is not only associated with the Aβ burden of the brain parenchyma, but also occasionally induces necrosis resulting in cerebral hemorrhage [174, 175]. However, the degree of CAA varies between AD brains, and the majority of AD patients showing microvascular amyloid deposits do not experience cerebral hemorrhage [176]. Nonetheless, because CAA could contribute to cortical dysfunction, regulation of vascular Aβ accumulation is suggested for mitigation of secondary AD pathology.

In human atherosclerosis, expression of NR4A including Nurr1 receptors is increased in macrophages after inflammatory stimulation [177]. In addition, overexpression of Nurr1 inhibited vascular lesion formation through reduction of smooth muscle cell proliferation and inflammatory response [135, 178]. These findings prove that endogenous Nurr1 suppresses macrophage activation, foam-cell formation, and further differentiation. Thus, it provides further evidence that atherogenesis could be prevented by modulation of Nurr1 expression. Therefore, Nurr1 may be proposed as a novel therapeutic target for preventing vascular disruption-related diseases mediated by AD.

### 2.8. The role of Nurr1 in metabolism

There are a number of studies describing both a correlation between type 2 diabetes and the risk of AD, and an association of hyperglycemia with AD pathophysiology [179, 180]. In addition, it has been reported that excess weight in middle age is related to increased risk of AD [181]. Metabolic hormones such as insulin, leptin, ghrelin, and adiponectin have been reported to have therapeutic potential and are also involved in the pathogenesis of AD [182]. Since AD could be accompanied by metabolic disturbances, normalization of metabolism will provide new insights into AD treatment.

There are several studies showing the roles of Nr4a receptors associated with metabolic functions. In particular, it has been reported that aberrant expression of Nurr1 is correlated to glucose metabolism and metabolic syndrome [183, 184]. Moreover, Nr4a receptors are expressed under metabolism-related stimuli including cold, fatty acids, glucose, insulin, and cholesterol. Therefore, it has been suggested that Nr4a receptors can be therapeutic targets for metabolism-related disorders [185]. There is also compelling evidence suggesting that Nr4a receptors show a potent association with the course of type 2 diabetes through regulation of insulin sensitivity and glucose homeostasis. Even though the mechanisms underlying the regulation of glucose metabolism by Nr4a receptors have not been elucidated, Nr4a receptors are identified as potential biological targets for diabetic patients [186]. However, the association of Nurr1 with carbohydrate metabolism is currently unclear. Therefore, identification of the correlation between Nurr1 and metabolic disorders in AD will be a novel pioneering field of study.

### 2.8. The role of Nurr1 in the habenula

The habenula is a part of the epithalamus in the diencephalon, located dorsal-medial to the posterior thalamus. It is divided into two regions: (1) the lateral habenula (LHb), which is innervated by the rostromedial tegmental nucleus (RMTg) to the dopamine-related regions, such as the ventral tegmental area (VTA) and substantia nigra (SN) or the serotonin system, such as the dorsal raphe nucleus and median raphe nucleus, and (2) the medial habenula (MHb), which is innervated with the serotonin system through the interpeduncular nucleus (IPN) [187-190]. The habenula, which is connected to the limbic system, basal ganglia, and pineal gland, is involved in the reward system and cognitive functions such as learning, memory, and attention [191]. Therefore, it has been suggested that the habenula may be involved in psychiatric disorders such as depression, schizophrenia, and drug-induced psychosis [192].

In adults and during development, the Nurr1 and Nr4a2 genes are robustly and specifically expressed in the MHb expressing both the choline acetyltransferase in the ventral part and the neuropeptide SP in the dorsal part [191, 193-195]. In addition, Nurr1 has been reported to mediate a gene pathway involved in habenula development regulated by the POU-domain transcription factor Brn3a [195]. Notably, several studies have suggested that habenular activity is involved in depression, which is one of the most common psychiatric symptoms in AD and a *risk factor for AD development*.

In the genetic helpless model and α-methyl-para-tyrosine-induced depression model, brain metabolism and glucose metabolism were elevated in the habenular compared to the control while metabolism of other brain regions was reduced [196, 197]. In patients with depression, it is shown that habenula activity is strongly correlated with dorsal raphé nuclei activity providing an evidence for the important roles of feedback pathway between habenula and dorsal raphé nuclei in controlling release of serotonin [198]. Application of deep brain stimulation to the LHb in a therapy-refractory patient with depression caused successful remission of depression, clarifying the relationship between the habenula and depression [199]. These reports suggest that upregulation of Nurr1 may have a positive effect on not only AD-related pathology but also the psychiatric symptoms that may occur in patients with AD.

## 3. Therapeutic potential of Nurr1 activation for AD treatment

As described so far, Nurr1 has the potential to mitigate the various pathophysiological consequences caused by AD. Nurr1 inhibits NF-κB signaling by binding to and clearance of NF-κB-p65 [79]. Therefore, it can be speculated that modulation of Nurr1 expression can suppress neuroinflammatory responses as well as beta-secretase 1 (BACE1), which is mediated by NF-κB signaling [200]. In addition, Nurr1 plays a neuroprotective role against neuronal death induced by various toxic mediators such as ROS and 1-methyl-4-phenyl-1,2,3,6-tetrahydropyridine (MPTP) [59, 60]. Furthermore, upregulation of Nurr1 has been reported to enhance cognitive function as well as increase hippocampal neurogenesis by enhancing the proliferation and differentiation of NPCs [18]. Considering the beneficial effects of Nurr1 enhancement on the pathological symptoms associated with AD, such as neuroinflammation, neuronal loss, impaired neurogenesis, and cognitive dysfunction, compensation and enhancement of the degenerated Nurr1 in AD patients may be a promising therapeutic target. Remarkably, one recent study reported that Nurr1 regulates AD-related pathogenesis and cognitive function in Aβ-overexpressing mice, supporting the therapeutic potential of Nurr1 for AD [16].

Interestingly, Nurr1 not only forms homo- or heterodimers with other members of the NR4A family, but can also forms heterodimers with RXRs via DR5 response element [201]. In addition, since the activation of nuclear receptors such as RXR, liver X receptor (LXR), and peroxisome proliferator-activated receptor (PPAR)-γ is known to affect Aβ generation and Aβ clearance, Nurr1 may contribute to the alleviation of Aβ-related pathophysiology through interactions with other nuclear receptors [202-205].

**Figure 1.**
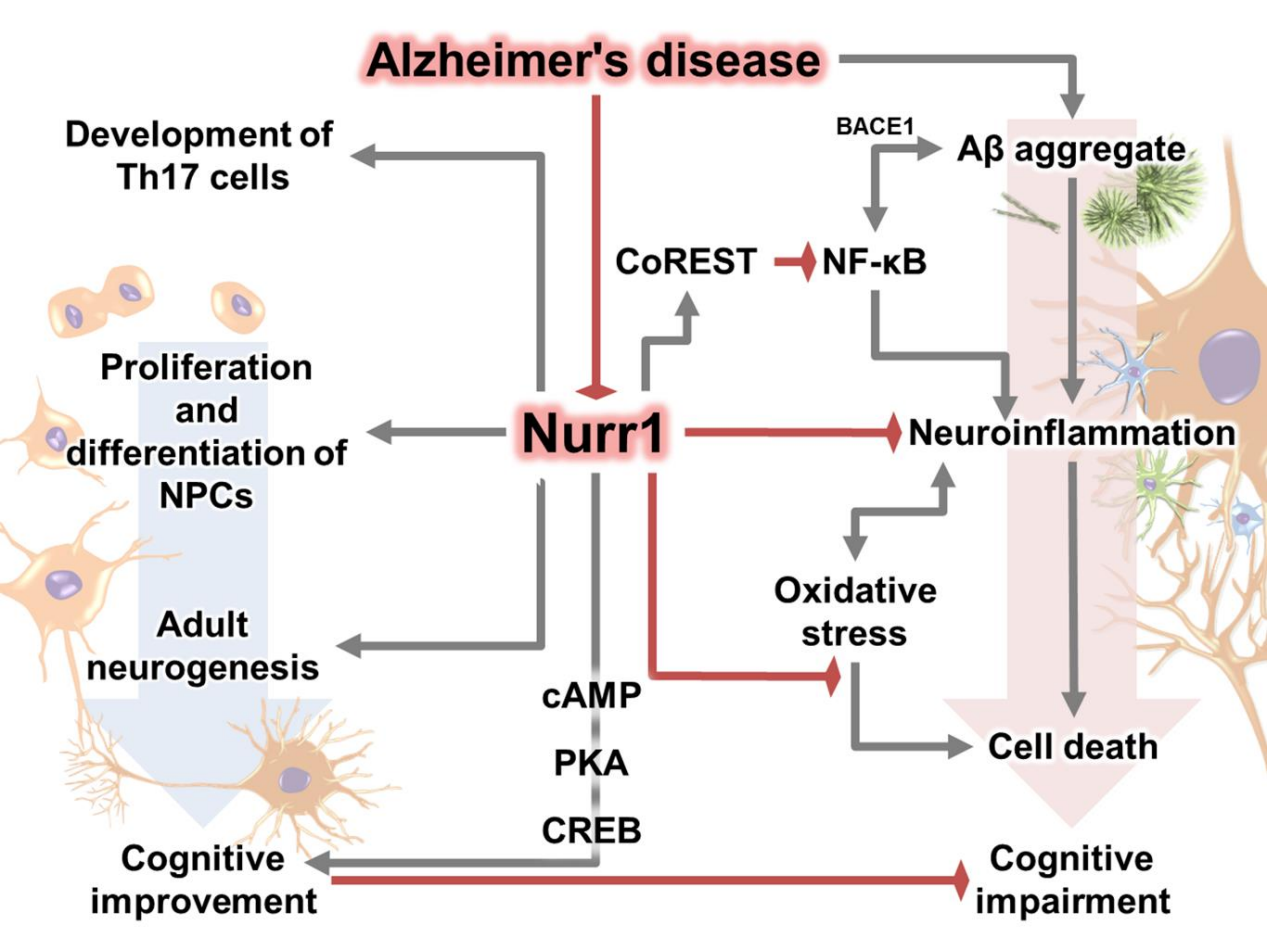
Overview of effect of Nurr1 in Alzheimer’s disease.

Therefore, many researchers have applied various methods to identify potential activators, ligands, and agonists of Nurr1 and have suggested several candidate structures [206-208]. To date, several Nurr1 agonists/activators have been identified and have shown positive effects in autoimmune disease and various PD models, suggesting the potential for the therapeutic effect of Nurr1 in neurodegenerative disease including AD [19, 20, 209-213]. Therefore, a breakthrough for AD treatment will be to prove the efficacy of Nurr1 agonists/mimetics or gene delivery of Nurr1 in animal experiments or clinical trials. A number of studies have suggested that modulation of Nurr1 function may serve as a great strategy to control AD pathogenesis (Fig. 1), and one recent study has shown a colocalization and correlation between Nurr1 and Aβ, and demonstrated that administration of Nurr1 agonists alleviates AD-related pathologies in Aβ-overexpressing mice [16].

## 4. Nurr1 and brain disorders

As mentioned earlier, Nurr1 can contribute to the beneficial effects on AD-associated pathologies and may have therapeutic potential for AD. Notably, there have been studies regarding the roles of Nurr1 in various conditions in related to AD pathologies. It is speculated that reviewing the roles of Nurr1 in these conditions could provide and extend insights about possible applications of Nurr1 in the modulation of AD pathogenesis. In the following sections, the roles of Nurr1 in brain disorders will be described.

### 4.1. Parkinson’s disease

Nurr1 is known to be a key regulator of the development and maintenance of dopaminergic neurons in the midbrain [7, 78, 214, 215] and plays an important role in inhibiting neuronal death through suppression of inflammatory gene expression in microglia and astrocytes [79, 216]. Therefore, numerous studies have investigated whether Nurr1 may be associated with the pathogenesis of PD, which results from the degeneration of midbrain dopaminergic neurons [217]. In addition to decreased expression of Nurr1 in postmortem brain tissue and the peripheral blood of PD patients, a functional mutation of Nurr1 was found in PD [13, 218-220]. Interestingly, activation of Nurr1 or the Nurr1:RXRα heterodimer by agonists such as AQ, SA00025, and BRF110 has been reported to diminish neuronal loss, neuroinflammation, and behavioral symptoms that occur in 6-hydroxydopamine or MPTP-induced PD models [19, 20, 221, 222]. Remarkably, it has been reported that Nurr1 expression is down-regulated in dopaminergic neurons with NFTs of the SN of AD patients. This results indicate that dysregulation of Nurr1 is associated with tauopathies in the dopaminergic neurons of AD patients [13]. In contrast, a recent study demonstrated that Nurr1 expression is not altered in the SN of postmortem brains of AD patients [16]. This inconsistency may be due to the presence of NFT in the neurons of AD brains. As Nurr1 expression levels were not altered in neurons without NFT [13], although the latter study did not provide further information regarding the presence of NFT in the neurons [16]. These data provide evidence that Nurr1 may be a relevant target for alleviating AD pathogenesis, especially in tauopathy.

### 4.2. Ischemic stroke

A recent study has shown that Nurr1 expression was dynamic following acute ischemia induced by middle cerebral artery occlusion (MCAO)/reperfusion in a rat model. There was a negative correlation between Nurr1 and infarct volume up to 12 hours after ligation, but a positive correlation was observed after 24 hours. Nurr1 overexpression inhibited tumor necrosis factor-α (TNF-α) levels in microglia. Increase of Nurr1 expression through suppression of miR-145-5p, a negative regulator of Nurr1 alleviated infarct volume and improved the neurological outcomes in an acute stroke model [223]. In addition, transplantation of Nurr1-overexpressing human embryonal carcinoma cells into the ischemic striatum restored the behavioral disorder in a transient MCAO rat model [153]. Considering that TNF-α released from Aβ-activated microglia is a key cytokine causing cell cycle events, which are related to pathogenesis of neuronal death in AD [224], these regulatory effects of Nurr1 on microglial activation could be a therapeutic target for AD as well as ischemic stroke.

### 4.3. Schizophrenia

Nurr1 heterozygous mice exhibited behavioral patterns associated with the symptoms of schizophrenia and were suggested as a potential animal model of schizophrenia [15, 51]. Furthermore, protein and mRNA expression levels of Nurr1 were reduced in the prefrontal cortex of schizophrenia patients [225]. Considering that the abnormal function of dopaminergic neurons in the cerebral cortex and subcortical areas is associated with schizophrenia [226], the changes in Nurr1 expression in schizophrenia address necessitate studies examining the correlation between schizophrenia and Nurr1. Despite that hyperactivation of dopaminergic neurotransmission, conventionally considered a major hypothesis for pathology schizophrenia, these findings may support the recent challenges against the conventional dopamine hypothesis [226]. In relation to changes in Nurr1 expression in AD, the reduced number of Nurr1-expressing cells in subiculum of AD model mice with disease progress [64] and decreased levels of dopamine in various regions including the hippocampus of Nurr1 heterozygous mice, a schizophrenic animal model [15], may demonstrate some similarities in molecular changes involving both disorders as well as provide additional insights for further studies comparing mechanisms between two diseases.

### 4.4. Addictive behaviors

Although the role of Nurr1 in addiction is controversial, depending on the duration of treatment and the drug used, a number of studies have reported that the administration of addictive drugs such as cocaine and heroin reduces Nurr1 transcript levels in the midbrain [227-230]. In addition, Nurr1 heterozygous mice exhibited reduced reward-seeking behaviors mediated by dopaminergic neurotransmission and were vulnerable to neuro-degeneration during long-term methamphetamine administration [14, 231]. In contrast, the hippocampus of ketamine-addicted rats has been reported to show increased levels of Nurr1 due to CREB-medicated phosphorylation [232, 233]. These studies suggest that Nurr1 is involved in the initiation and progression of addictive disorders, which may depend on the type of drug and the duration of administration. As several lines of evidence suggest that reward processing is defective in neurodegenerative diseases including AD [234], the correlation between Nurr1 expression and addictive behaviors may suggest the need for further investigation regarding the relationship between Nurr1 function in AD and addictive behaviors.

### 4.5. Attention deficit hyperactivity disorder (ADHD)

VTA, a dopamine nucleus brain region with robust expression of Nurr1 [235, 236], projects dopamine axons to the prefrontal cortex [237], and dopamine system is considered to be an important part of ADHD pathogenesis [238, 239]. In addition, decreased dopamine synaptic markers have been reported in the dopamine reward pathway in ADHD patients [240]. An *in vivo* study using Nurr1 knockout mice with prenatal immune activation as an attention impairment model reported that genetic and environmental factors synergistically affected attentional impairment as well as additively affected locomotor hyperactivity. Remarkably, Nurr1 heterodeficient mice showed increased locomotor activity, and exhibited altered inflammatory cytokine responses against prenatal immune activation [241]. Although there were inconsistent reports regarding the levels of cytokines in AD, cytokines such as IL-6 and IL-10 are known to play important roles in AD pathogenesis [242]. Given that two NR4A2 polymorphisms were found in patients with ADHD [243], further studies investigating the changes of Nurr1 expression in ADHD may provide a better understanding of AD pathogenesis.

### 4.6. Circadian rhythm disorder

Disorders of the midbrain dopaminergic neurons, which are the basis of the reward system in the brain, are involved in the disruption of the circadian rhythm [244]. Notably, sleep and circadian rhythm disorder are early biomarkers of AD [245, 246]. The circadian nuclear receptor REV-ERBα encoded by the NR1D1 gene, competes with Nurr1 for the regulation of circadian TH expression via a target-dependent antagonistic mechanism [247]. In 6-month old 3xTg-AD mice, an animal model of AD, gene expression of NR1D1 is increased in the brainstem after exposure to darkness, compared to control mice [248]. Thus, these data may imply that controlling the balance between expression of ERV-ERBα and Nurr1 could be a potential target for treating circadian rhythm disorder in AD.

## 5. Conclusion

Recent findings regarding the Nurr1 role in the CNS have demonstrated molecular, cellular, and physiological responses underlying various conditions, and these findings may provide insights for the association between Nurr1 and the underlying mechanisms of AD (Fig. 1). Studies on the effect of Nurr1 support the correlation between Nurr1 expression and various stages of AD pathology and symptoms, including neuronal cell death, inflammation, synaptic loss, impaired adult neurogenesis, psychiatric symptoms, and cognitive deficits.

All mechanisms of development of neuro-degenerative diseases, especially of AD, are closely related to the actions of Nurr1. Nurr1 may be capable of regulating AD-related pathogenesis, based on recent studies showing the critical roles of Nurr1 in AD-related pathology (Fig. 1). As a result, subsequent experiments have been performed to prove Nurr1 as a potential target for treatment of AD, and have suggested Nurr1 agonists/mimetics as potential therapeutic agents for AD.
